# Supplementary material for: Complexome profiling of the Chlamydomonas psb28 mutant reveals TEF5 as an early PSII assembly factor
Source: Plant Cell. 2025 Mar 17;37(6):koaf055. doi: 10.1093/plcell/koaf055 (PMC12164590; doi:10.1093/plcell/koaf055)
Supplement: koaf055_Supplementary_Data [file koaf055_supplementary_data.zip › psb28_interactive.zip!/plots/intensity/qualityPlots/peptides/ATPG_Cre11.g481450.t1.2.html]

### Cre11.g481450.t1.2

Trivial name: ATPG  
SeqCoverage [median]: 56.90  
Mass: 22.48 kDa  
  
 

| Sequence | MissedCleavage | Mass [Da] | wtIntensity | muIntensity | Score |
| --- | --- | --- | --- | --- | --- |
| AKITAEVESSIAGLEQESASMLK | 1 | 2391.22 | 19577.43 | 0.00 | 151.06 |
| DNTGDVDK | 0 | 862.37 | 0.00 | 373.82 | 107.69 |
| DNTGDVDKLVLEAETILK | 1 | 1972.04 | 219484.14 | 104458.99 | 233.79 |
| ISAEVLK | 0 | 758.45 | 19662.36 | 9318.52 | 128.86 |
| ISAEVLKR | 1 | 914.55 | 63415.77 | 38919.12 | 156.40 |
| ITAEVESSIAGLEQESASMLK | 0 | 2192.09 | 140046.43 | 180055.62 | 231.45 |
| KAAKQSELDKTYNEAK | 3 | 1822.94 | 0.00 | 711.99 | 67.42 |
| LGSVKDNTGDVDK | 1 | 1346.67 | 2487.71 | 2075.91 | 107.46 |
| LGSVKDNTGDVDKLVLEAETILK | 2 | 2456.34 | 40048.89 | 35903.66 | 160.57 |
| LVLEAETILK | 0 | 1127.68 | 10064.48 | 4016.04 | 107.15 |
| QSELDK | 0 | 718.35 | 0.00 | 3478.18 | 86.51 |
| QSELDKTYNEAK | 1 | 1424.68 | 416286.72 | 292985.64 | 156.73 |
| SDVSAMINTK | 0 | 1064.52 | 519926.11 | 444173.92 | 261.60 |
| SDVSAMINTKK | 1 | 1192.61 | 60856.72 | 80059.20 | 150.33 |
| SKLGSVK | 1 | 717.44 | 0.00 | 10006.95 | 125.58 |
| SKLGSVKDNTGDVDKLVLEAETILK | 3 | 2671.46 | 2697.54 | 0.00 | 35.92 |
| SLDAQVDK | 0 | 874.44 | 176293.58 | 106300.42 | 223.87 |
| SLDAQVDKISAEVLK | 1 | 1614.88 | 45092.39 | 38721.71 | 155.14 |
| SLDAQVDKISAEVLKR | 2 | 1770.98 | 549716.02 | 841068.22 | 261.59 |
| TWFTPVGK | 0 | 934.49 | 91716.39 | 80555.30 | 139.68 |
| VLDERDNLIR | 1 | 1241.67 | 608031.97 | 623886.83 | 140.45 |
| VLPEGVR | 0 | 768.45 | 35919.65 | 13563.19 | 103.29 |
| VLPEGVRV | 1 | 867.52 | 200914.40 | 413248.81 | 139.31 |
|  |  |  | 3222238.7 | 3323882.0 |  |
